# Cuproptosis-related genes predict prognosis and trastuzumab therapeutic response in HER2-positive breast cancer

**DOI:** 10.1038/s41598-024-52638-8

**Published:** 2024-02-05

**Authors:** Rui Sha, Xinrui Dong, Shanshan Yan, Huijuan Dai, Aijun Sun, Liuxia You, Zongjin Guo

**Affiliations:** 1 Department of Thyroid and Breast Surgery, The First Affiliated Hospital of Wannan Medical College (Yijishan Hospital of Wannan Medical College), 2 Zheshan West Road, Wuhu, 241001 Anhui China; 2 Department of Breast Surgery, The First Affiliated Hospital With Nanjing Medical University, 300 Guangzhou Road, Nanjing, 210029 Jiangsu China; 3 Center for Medical Ultrasound, Suzhou Municipal Hospital, Nanjing Medical University Affiliated Suzhou Hospital, Suzhou, 215000 Jiangsu China; 4 Renji Hospital, School of Medicine, Shanghai Jiaotong University, 1630 Dongfang Road, Shanghai, 200127 China; 5 Department of Thyroid and Breast Oncological Surgery, Xuzhou Medical College Affiliated Huaian Hospital, 62 Huaihai South Road, Huaian, 223001 Jiangsu China; 6 Department of Clinical Laboratory, The Second Affiliated Hospital of Fujian Medical University, Quanzhou, 362000 Fujian China; 7 Department of Interventional Radiology, The University of HongKong-Shenzhen Hospital, Shenzhen, 518053 China

**Keywords:** Predictive markers, Breast cancer, Tumour immunology

## Abstract

Breast cancer is the most common diagnosed cancer, the HER2-positive subtype account for 15% of all breast cancer. HER2-targeted therapy is the mainstay treatment for HER2-positive breast cancer. Cuproptosis is a novel form of programmed cell death, and is caused by mitochondrial lipoylation and destabilization of iron-sulfur proteins triggered by copper, which was considered as a key player in various biological processes. However, the roles of cuproptosis-related genes in HER2-positive breast cancer remain largely unknown. In the present study, we constructed a prognostic prediction model of HER2-positive breast cancer patients using TCGA database. Dysregulated genes for cells resistant to HER2-targeted therapy were analyzed in the GEO dataset. KEGG pathway, GO enrichment and GSEA was performed respectively. The immune landscape of DLAT was analyzed by CIBERSORT algorithm and TIDE algorithm. HER2-positive breast cancer patients with high CRGs risk score showed shorter OS. DLAT was downregulated and correlated with better survival of HER2-positive breast cancer patients (HR = 3.30, *p* = 0.022). High expressed DLAT was associated with resistant to HER2-targeted therapy. Knocking down DLAT with siRNA increased sensitivity of breast cancer to trastuzumab. KEGG pathway and GO enrichment of DEGs indicated that DLAT participates in various pathways correlated with organelle fission, chromosome segregation, nuclear division, hormone-mediated signaling pathway, regulation of intracellular estrogen receptor signaling pathway, condensed chromosome and PPAR signaling pathway. There was a negative correlation between TIDE and DLAT expression (r = − 0.292, *p* < 0.001), which means high DLAT expression is an indicator of sensitivity to immunotherapy. In conclusion, our study constructed a four CRGs signature prognostic prediction model and identified DLAT as an independent prognostic factor and associated with resistant to HER2-targeted therapy for HER2-positive breast cancer patients.

## Introduction

Breast cancer (BC) has become the most frequently diagnosed cancer, and it is also the leading cause of cancer-related death in women worldwide^1,2^. BC can be divided into four conventional subtypes, Luminal A, Luminal B, human epidermal growth factor 2 (HER2) positive and triple negative breast cancer (TNBC)^3^. Endocrine therapy is used for individuals with hormone receptor positive disease, chemotherapy is the primary treatment option for TNBC, and HER2-targeted therapy is utilized for patients whose tumors overexpress HER2^4,5^.

About 15% of patients developed HER2-positive BC, which was characterized by a high recurrence rates, aggressive phenotype, and poor survival outcomes^6,7^. The monoclonal antibody trastuzumab significantly improves the prognosis of HER2-positive individuals^8–10^. Over the past 20 years, significant progress has been achieved in the treatment of HER2-positive BC, and the U.S. Food and Drug Administration (FDA) has approved 7 kinds of HER2-targeted therapies for the treatment of early and/or advanced disease^11^. These treatment advances have converted into higher cure rates for early-stage disease, as well as significantly longer progression free survival (PFS) and overall survival (OS) in metastatic patients^12^. Despite these advances, HER2-positive metastatic BC remains an almost invariably fatal disease, and the efficacy of a single HER2-targeted therapy is transient, particularly for patients who relapse after neoadjuvant therapy containing monoclonal antibody, with median PFS of approximately 1 year and less than 1 year in the first and second lines, respectively^13^. While primary resistance to HER2-targeted therapy is possible, most treatment failures stem from acquired resistance to cell subclones gradually selected over the course of treatment. Primary or secondary resistance to HER2-targeted therapy, however, remains a vexing problem^11,14^. In fact, BC is a heterogeneous disease with distinct molecular features, biological characteristics and clinical outcomes^15,16^. In this context, treatments that can induce other forms of regulated cell death (RCD), including apoptosis, ferroptosis, pyroptosis, necroptosis, have emerged as potential strategies to treat drug-resistant BC and improve survival^17,18^.

Cuproptosis is a recently identified RCD, which is a process occurs via direct binding of copper to lipoylated components of the tricarboxylic acid (TCA) cycle^19^. The intracellular copper concentration is controlled by copper importers *SCL31A1* and copper exporters *ATP7A* and *ATP7B*. In addition, serum copper concentrations in BC patients are higher than that in healthy controls and patients with benign breast disease, suggesting that elevated serum copper levels may be associated with an increased risk of BC^20^. In many other kinds of cancers, like lung cancer^21^, colorectal cancer^22^, thyroid cancer^23^ and prostate cancer^24^, copper concentrations are also higher than in healthy people. There is increasing evidence suggest that copper as involved in cancer development and spreading (epithelial to mesenchymal transition, angiogenesis), and the responsiveness of many copper complexes to cancer cells has been studied over the past four decades, many of which may become drug options for cancer treatment^25^. There are 14 cuproptosis-related genes (CRGs) so far. Since the concept of cuproptosis was proposed, CRGs have been considered valuable in predicting prognosis and immune infiltration in patients with BC^26–28^. However, the predictive value may vary among subtypes of BC. Based on CRGs, TNBC patients can be divided into two subtypes, which have significantly different immune invasion characteristics and tumor signaling pathways^29^. But the studies on the role of cuproptosis in HER2-positive BC lack to some extent.

In our study, we explored the correlation between CRGs with the prognosis of HER2-positive BC through accessing and analyzing the public database, and the prognostic gene set model was constructed. DLAT was identified as a CRGs associated with resistant to HER2-targeted therapy and immunotherapy sensitivity.

## Results

### Expression determination and survival assessment of cuproptosis-related genes in HER2-positive BC

**Figure 1:**
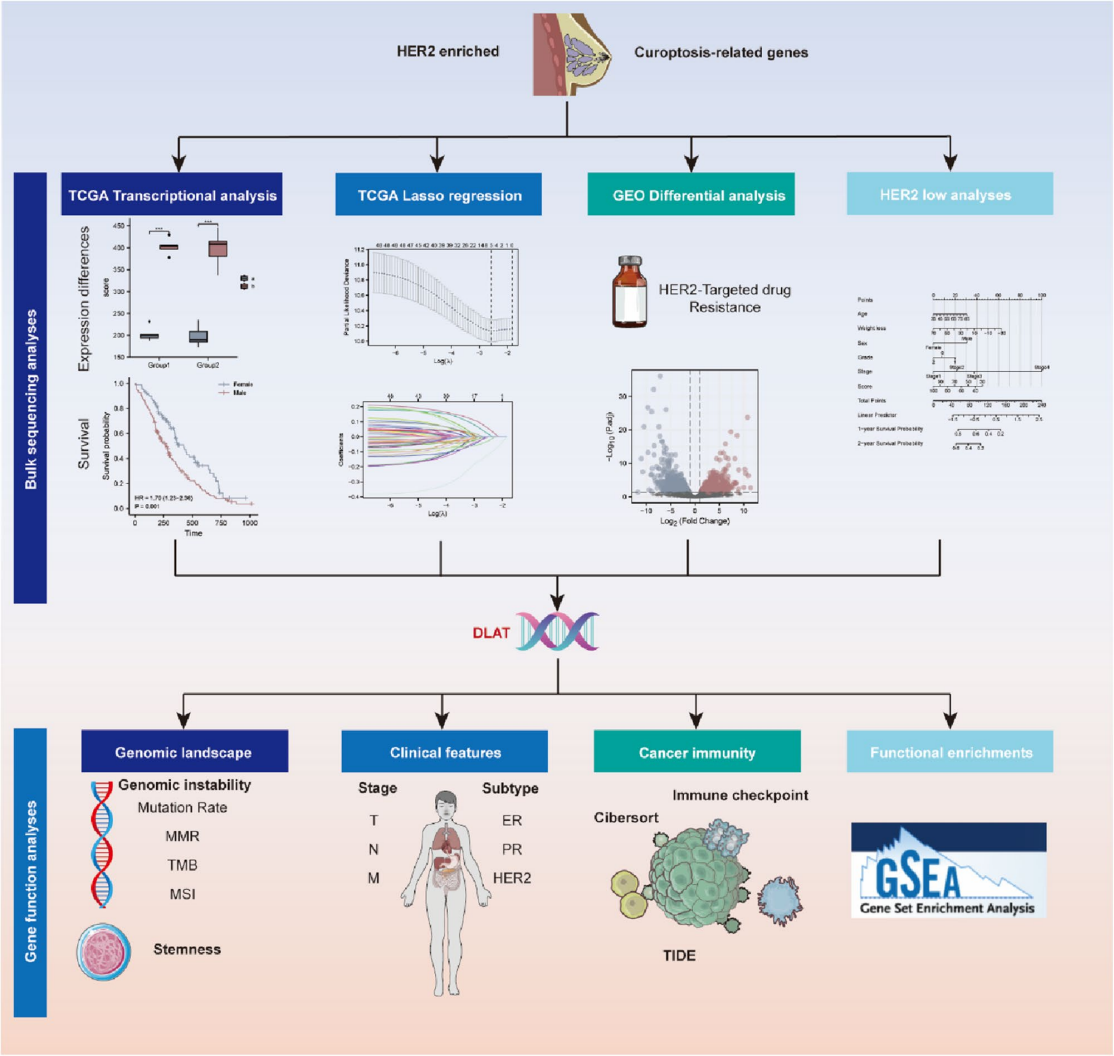
Workflow of the current study. All of the TCGA BRCA data used in this study are HER2-positive type, unless otherwise stated.

**Figure 2:**
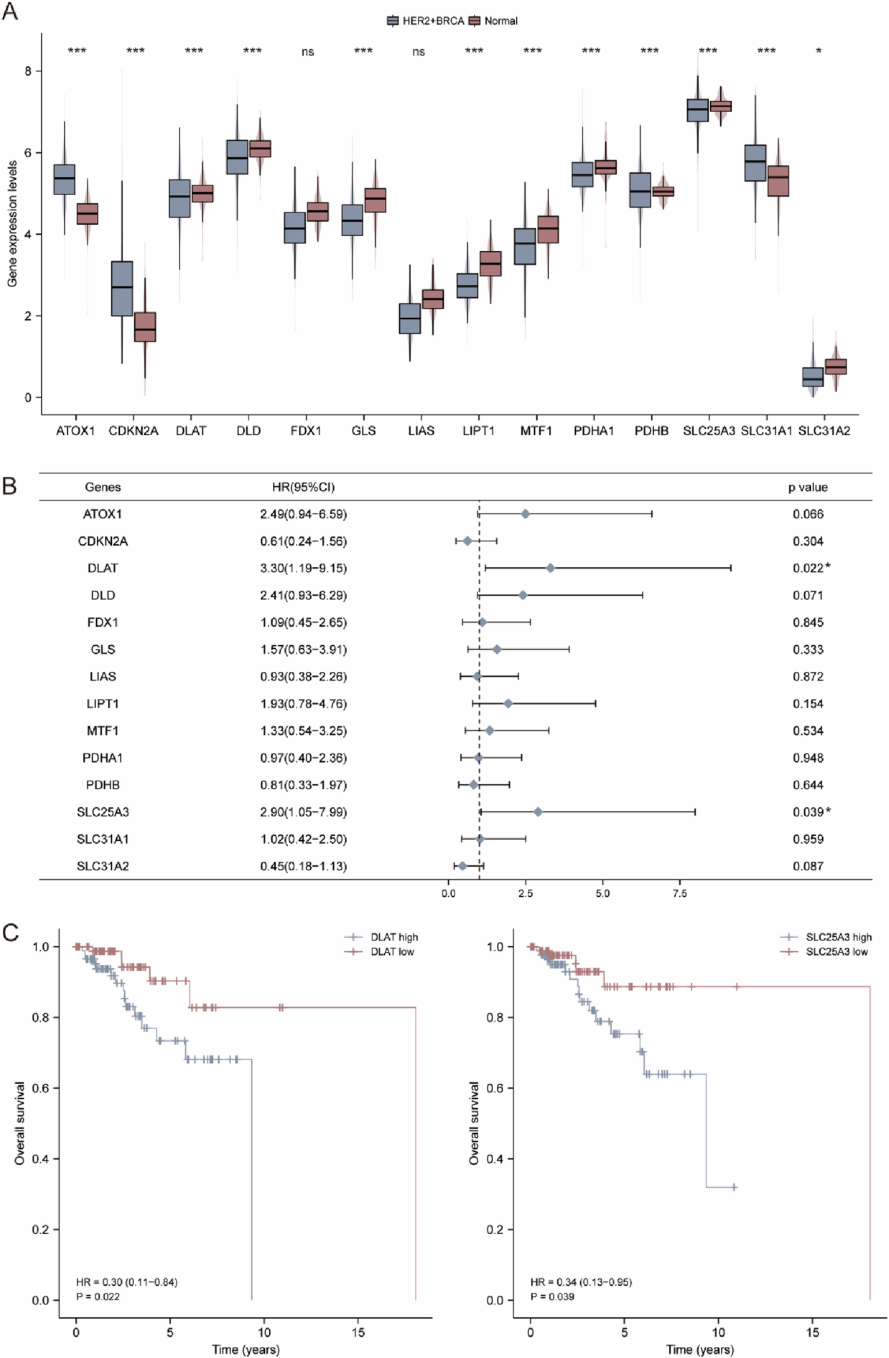
Expression difference and survival analysis of CRGs in HER2-positive breast cancer. (**A**) Expression difference of 14 CRGs in HER2-positive breast cancer and normal breast tissues. *p < 0.05; ***p < 0.001; ns, no significance. (**B**) The forest plot of univariate COX hazard model of the 14 CRGs. *p < 0.05. (**C**) The OS curves of *DLAT* and *SLC25A3* in patients with HER2-positive breast cancer in the low and high expression group.

The workflow of the study design was presented in Fig. 1. In this study, we identified 14 genes related to cuproptosis according to the literature^30^. The expression level of the 14 genes in HER2-positive BC were determined by the TCGA database. As shown in Fig. 2A, nine genes including *DLAT, DLD, GLS, LIPT1, MTF1, PDHA1, PDHB, SLC25A3* and *SLC31A2* were significantly downregulated, but the other 3 genes including *ATOX1, CDKN2A* and *SLC31A1* were significantly upregulated in HER2-positive BC compared with normal tissues. For *FDX1* and *LIAS*, however, there had no statistical difference between HER2-positive BC and normal breast tissues. Subsequently, the prognostic values of the 14 genes were evaluated in HER2-positive BC patients. The univariate Cox hazard model suggested a higher risk of death in the high expression of *DLAT* (HR = 3.30, *p* = 0.022) and *SLC25A3* (HR = 2.90, *p* = 0.039) group (Fig. 2B). The cumulative OS curves provided a visualization of the prognosis with different *DLAT* and *SLC25A3* expression (Fig. 2C).

### The prediction model for HER2-positive BC

**Figure 3:**
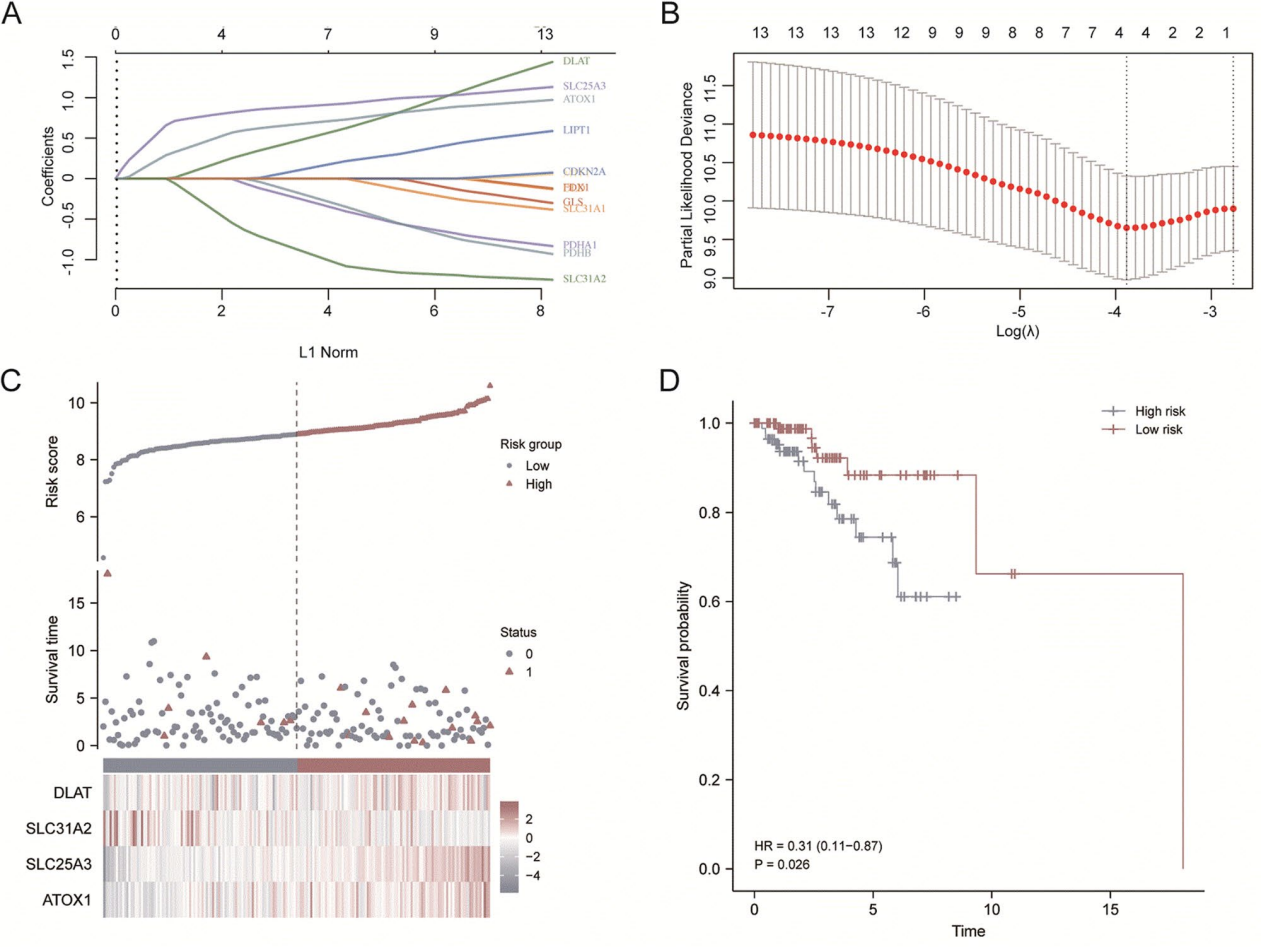
Construction of the cuproptosis-related prognostic signature. (**A**) LASSO regression analysis of 14 prognostic CRGs. (**B**) Cross validation method to select optimal genes. (**C**) Rank dot, scatter plots and heat map of the model gene expression. (**D**) Kaplan–Meier analyses of the OS between the high risk and low risk group.

Given that CRGs had distinct expression between normal breast tissues and HER2-positive BC, and part of these genes had predictive value for the prognosis of HER2-positive BC patients. We attempted to construct the prognostic gene set for HER2-positive BC using the LASSO method. The prognostic model was established using the 14 cuproptosis related genes in HER2-positive BC patients (Fig. 3A, B). The risk score = (0.1843) * *DLAT* + (− 0.4056) * *SLC31A2* + (0.7899) * *SLC25A3* + (0.4987) * *ATOX1*. The risk score, survival status and expression of patients with HER2-positive BC were shown in Fig. 3C. The further OS curve analysis showed that patients in the low-risk group had a lower risk of death and longer survival time (HR = 0.31, *p* = 0.026, Fig. 3D).

### *DLAT* is the potential gene resistant to HER2-targeted therapy

**Figure 4:**
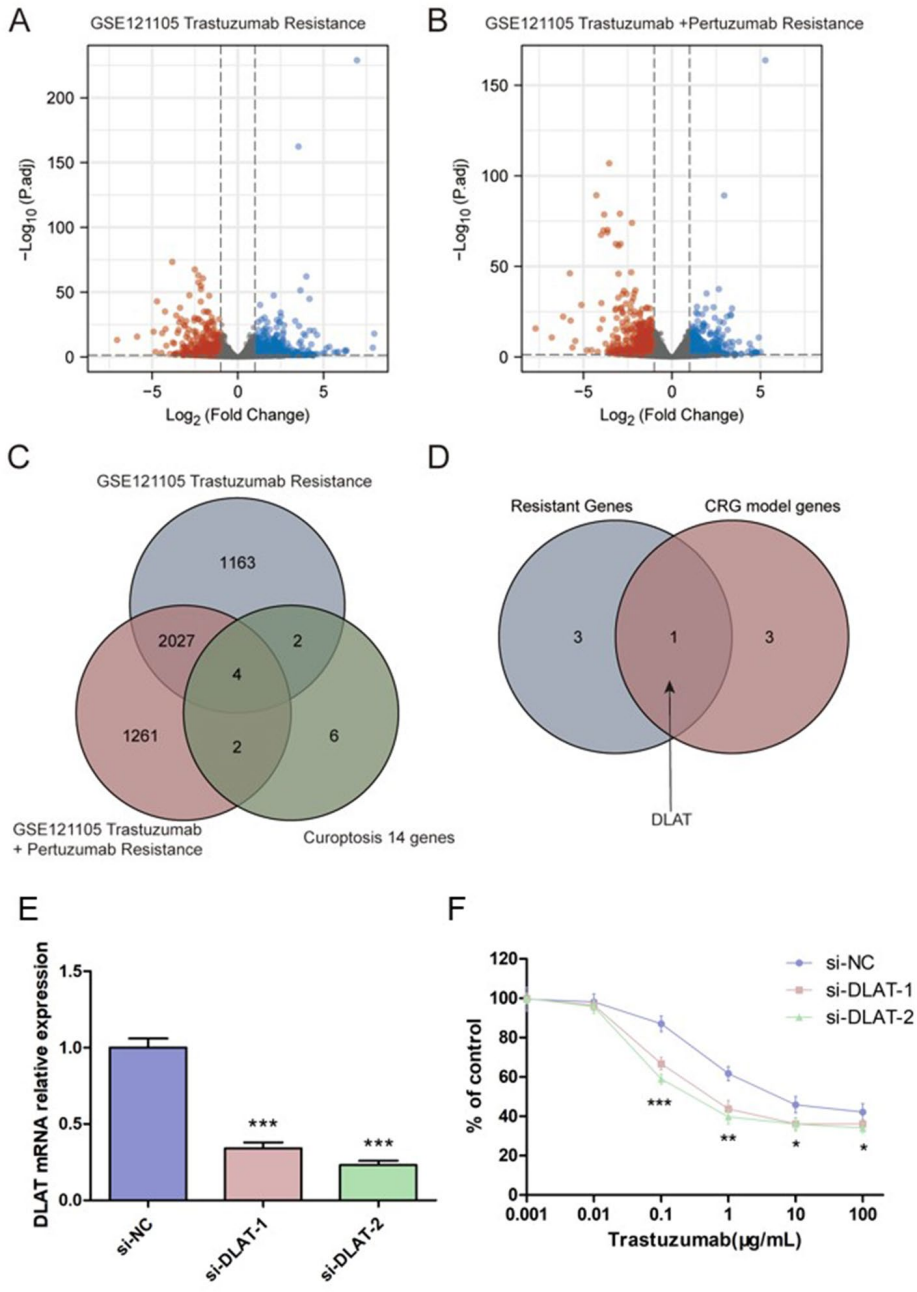
Selection for the CRGs that related to HER2-targeted therapy. (**A**) Dysregulated genes for cells resistant to trastuzumab in GSE121105. (**B**) Dysregulated genes for cells resistant to trastuzumab + pertuzumab in GSE121105. (**C**) Venn diagram of trastuzumab resistance, trastuzumab + pertuzumab resistance and CRGs. (**D**) Venn diagram of resistant genes and CRG model genes. (**E**) The relative expression of DLAT after transfected with si-DLAT. ***p < 0.001. (**F**) Effect of trastuzumab on growth inhibition in SKBR-3 breast cancer cells transfected with si-DLAT. *p < 0.05; **p < 0.01; ***p < 0.001.

To identify whether the CRGs have the predictive value for resistance to HER2-targeted therapy, we first analyzed the GEO dataset, GSE121105^31^. Dysregulated genes for cells resistant to trastuzumab or trastuzumab + pertuzumab in combination were showed in Fig. 4A, B, respectively. Compared with the 14 cuproptosis related genes, only 4 genes showed differently expression in all 3 datasets (Fig. 4C). Further analysis revealed that *DLAT* was the only gene included both in CRG model genes and resistant genes (Fig. 4D). RT-qPCR found that DLAT expression was significantly down-regulated after transfected with siRNA targeting DLAT (Fig. 4E). MTT assay revealed that DLAT silencing sensitized the SKBR-3 cell to trastuzumab (Fig. 4F).

### The relationship between *DLAT* expression and genetic characteristic of BC patients

**Figure 5:**
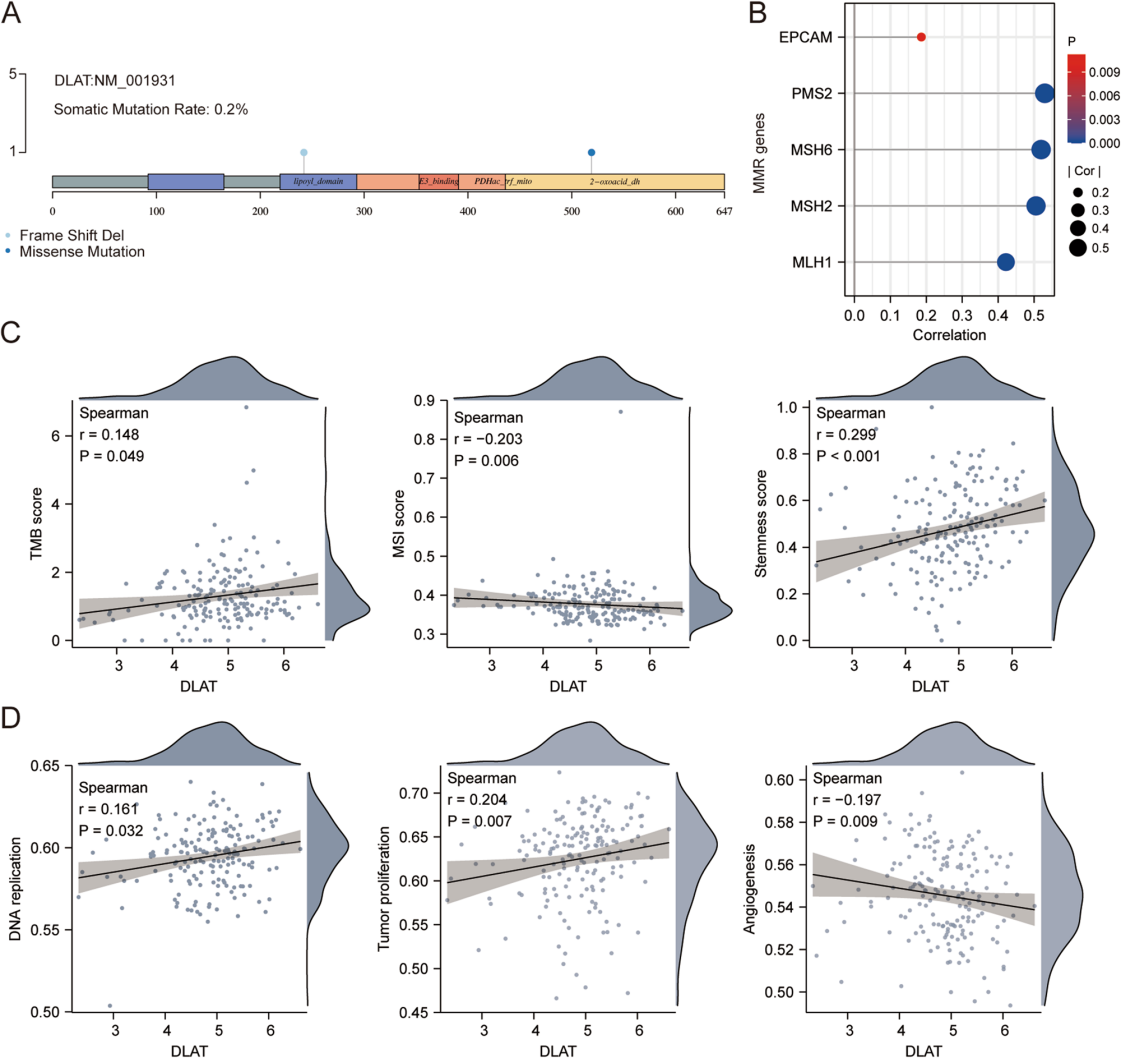
The genetic characteristic of *DLAT*. (**A**) The somatic mutation of *DLAT*. (**B**) The relationship between *DLAT* and MMR related genes. (**C**) The relationship between *DLAT* and TMB, TSI, stemness score. (**D**) The relationship between *DLAT* and DNA replication, tumor proliferation, angiogenesis.

The genetic characteristic of *DLAT* was identified. The somatic mutation rate of *DLAT* was 0.2% (Fig. 5A). The relationship between *DLAT* and MMR related genes was showed in Fig. 5B, and *DLAT* has significant correlation with *EPCAM, PMS2, MSH6, MSH2* and *MLH1*. *DLAT* also had significant correlation with TMB score (r = 0.148, *p* = 0.049), MSI score (r =  − 0.203, *p* = 0.006) and stemness score (r = 0.299, *p* < 0.001) (Fig. 5C). By using the ssGESA method, we identified *DLAT* had positive correlation with DNA replication (r = 0.161, *p* = 0.032) and tumor proliferation (r = 0.204, *p* = 0.007), however, *DLAT* had negative correlation with angiogenesis (r =  − 0.197, *p* = 0.009) (Fig. 5D).

### The relationship between *DLAT* expression and clinical characteristic of BC patients

**Figure 6:**
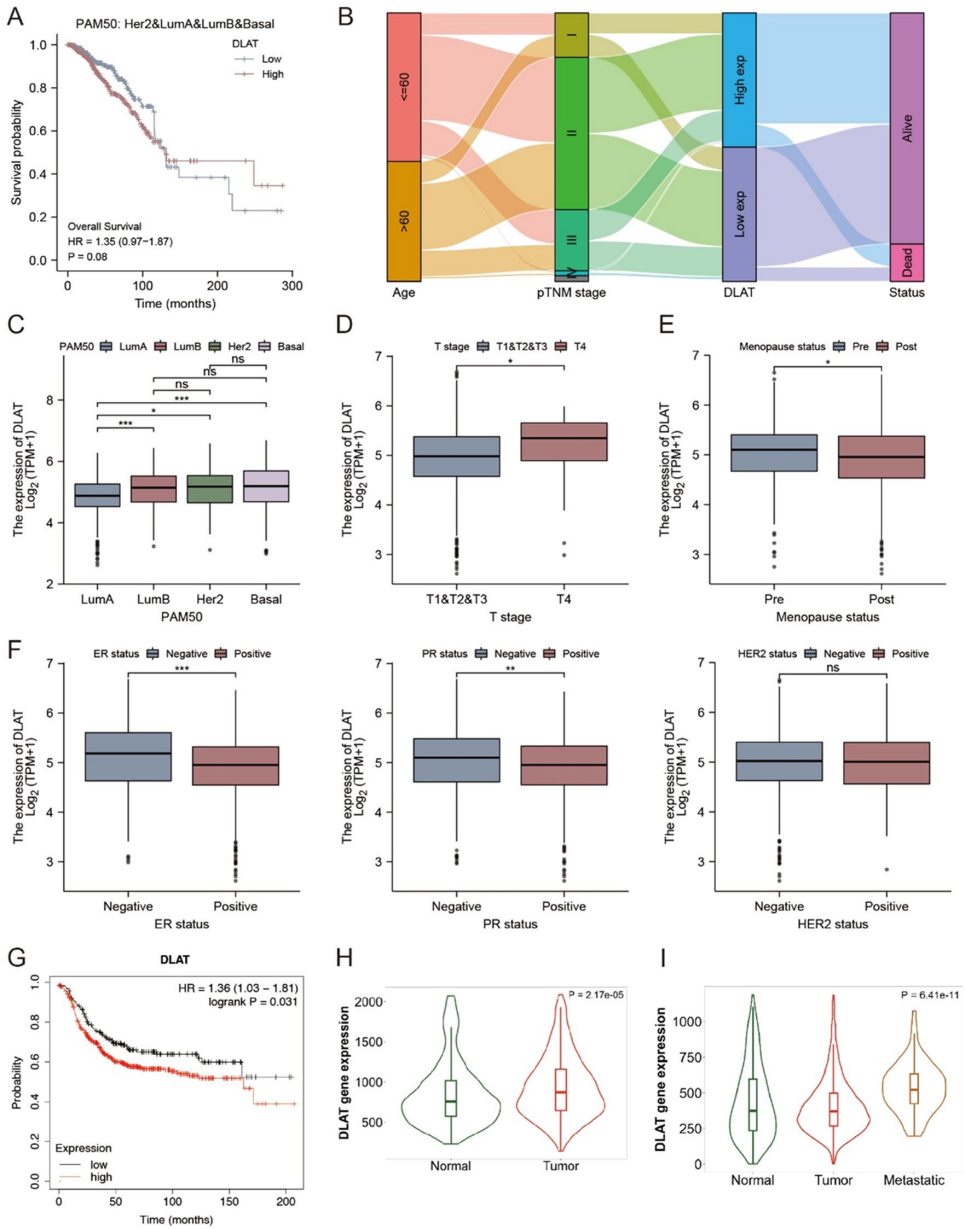
The clinical characteristic of *DLAT*. (**A**) The OS curves of breast cancer patients with *DLAT* low and high expression group. (**B**) The alluvial diagram of *DLAT* with clinical characteristic. (**C**) *DLAT* expression in different PM50 subtype. *p < 0.05; ***p < 0.001; ns, no significance. (**D**) *DLAT* expression in different T stage. *p < 0.05. (**E**) *DLAT* expression in different menopause status. *p < 0.05. (**F**) *DLAT* expression in different ER status, PR status and HER2 status. **p < 0.01; ***p < 0.001; *ns* No significance. (**G**) The RFS curves of HER2-positive BC patients with *DLAT* low and high expression group. (**H**) DLAT expression between normal breast tissues and HER2-positive BC tissues. (**I**) DLAT expression among normal breast tissues, HER2-positive BC tissues and metastatic tissues.

We analyzed the association between prognosis and *DLAT* expression in BC patients, however, there had no difference of OS between high or low *DLAT* expression patients (HR = 1.35, *p* = 0.08, Fig. 6A). To visualize the attributes of individual patients and display any correlations with survival, we constructed the alluvial diagram (Fig. 6B). The expression level of *DLAT* in different BC subtype according the PM50 was showed in Fig. 6C, the *DLAT* had lower expression in Luminal A than Luminal B (*p* < 0.001), HER2-positive (*p* < 0.05) and Basal subtype (*p* < 0.001), while there had no significant difference among Luminal B, HER2-positive and Basal subtype. *DLAT* had higher expression in T4 than T1&T2&T3 subtype (*p* < 0.05, Fig. 6D) and higher expression in pre-menopause than post-menopause patients (*p* < 0.05, Fig. 6E). *DLAT* also had higher expression in ER negative (*p* < 0.001) and PR negative patients (*p* < 0.01), however, there had no difference between HER2-negative and HER2-positive patients (Fig. 6F). In the KM-plotter database, patients in the *DLAT* low expression group (HR = 1.36, *p* = 0.031) had significantly longer RFS than patients in the high expression group (Fig. 6G). In the TNM-plotter database, the expression of DLAT had significantly difference between HER2-positive BC and normal breast tissues (*p* < 0.001, Fig. 6H), and it also varies among normal breast tissues, tumor tissues and metastatic tissues (*p* < 0.001, Fig. 6I).

### GO, KEGG and GESA analysis for *DLAT*

**Figure 7:**
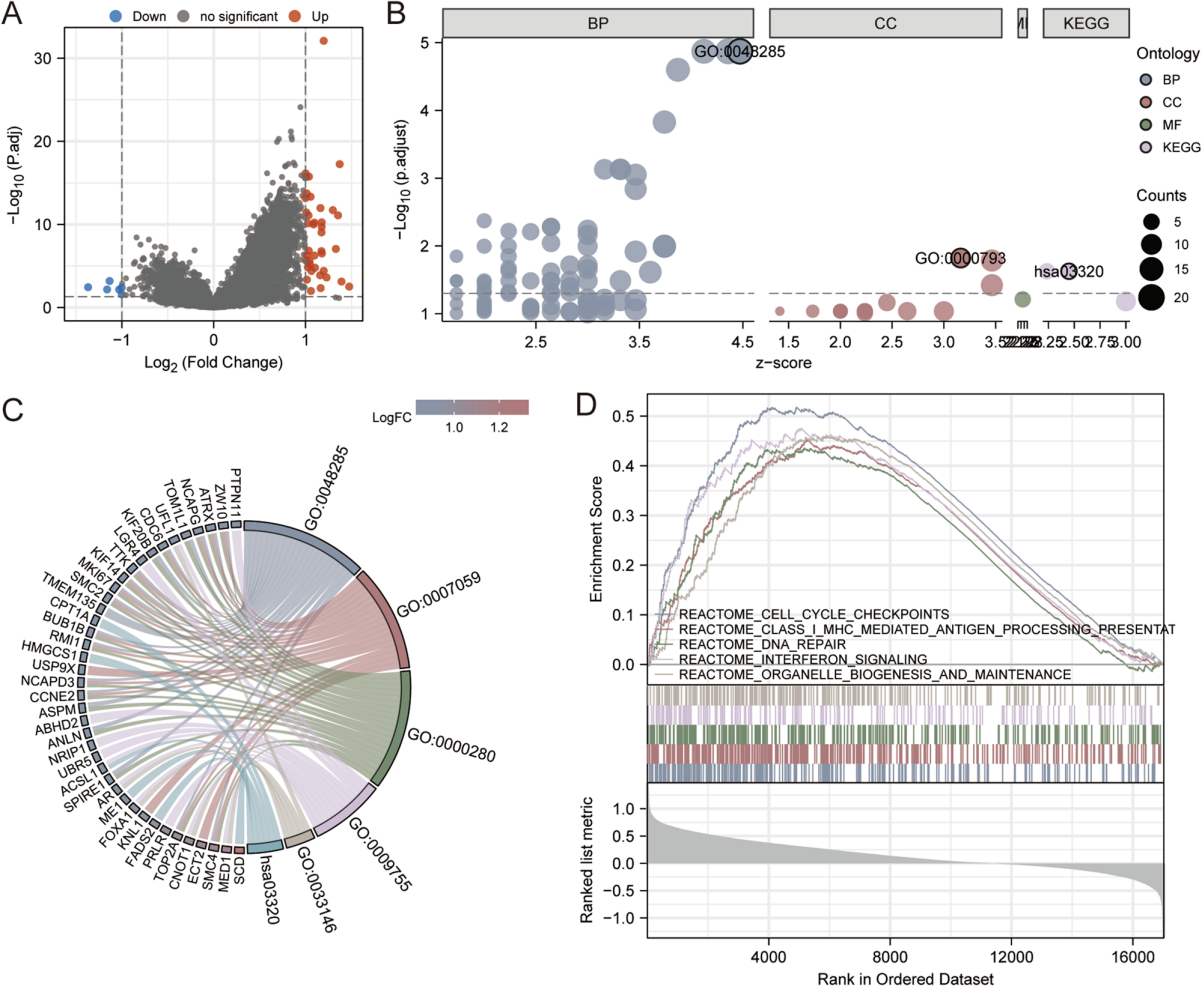
Functional analysis for *DLAT*. (**A**) The volcano map for DEGs in *DLAT* high or low group. (**B**) The GO enrichment and KEGG pathway for DEGs in *DLAT* high or low group. (**C**) The netplot of KEGG pathway of the DEGs. (**D**) The significantly enrichment pathway in *DLAT* high or low group according to the GSEA.

To further investigate the potential role of *DLAT* for HER2-positive BC, we divided the HER2-positive BC patients into two groups according to the *DLAT* expression, namely, *DLAT* high group and *DLAT* low group. The upregulated and downregulated genes between the two groups were showed in Fig. 7A, under the condition of given threshold of FDR < 0.05 and fold change > 1. KEGG pathway and GO enrichment of DEGs indicated that *DLAT* participates in various pathways correlate with organelle fission (GO:0048285), chromosome segregation (GO:0007059), nuclear division (GO:0000280), hormone-mediated signaling pathway (GO:0009755), regulation of intracellular estrogen receptor signaling pathway (GO:0033146), condensed chromosome (GO:0000793) and PPAR signaling pathway (hsa03320) (Fig. 7B, C). By performing GSEA, we further verified the potential roles of *DLAT* associated molecules in the regulation of cell cycle checkpoints, class I MHC mediated antigen processing presentation, DNA repair, interferon signaling and organelle biogenesis and maintenance (Fig. 7D).

### The immune landscape for *DLAT*

**Figure 8:**
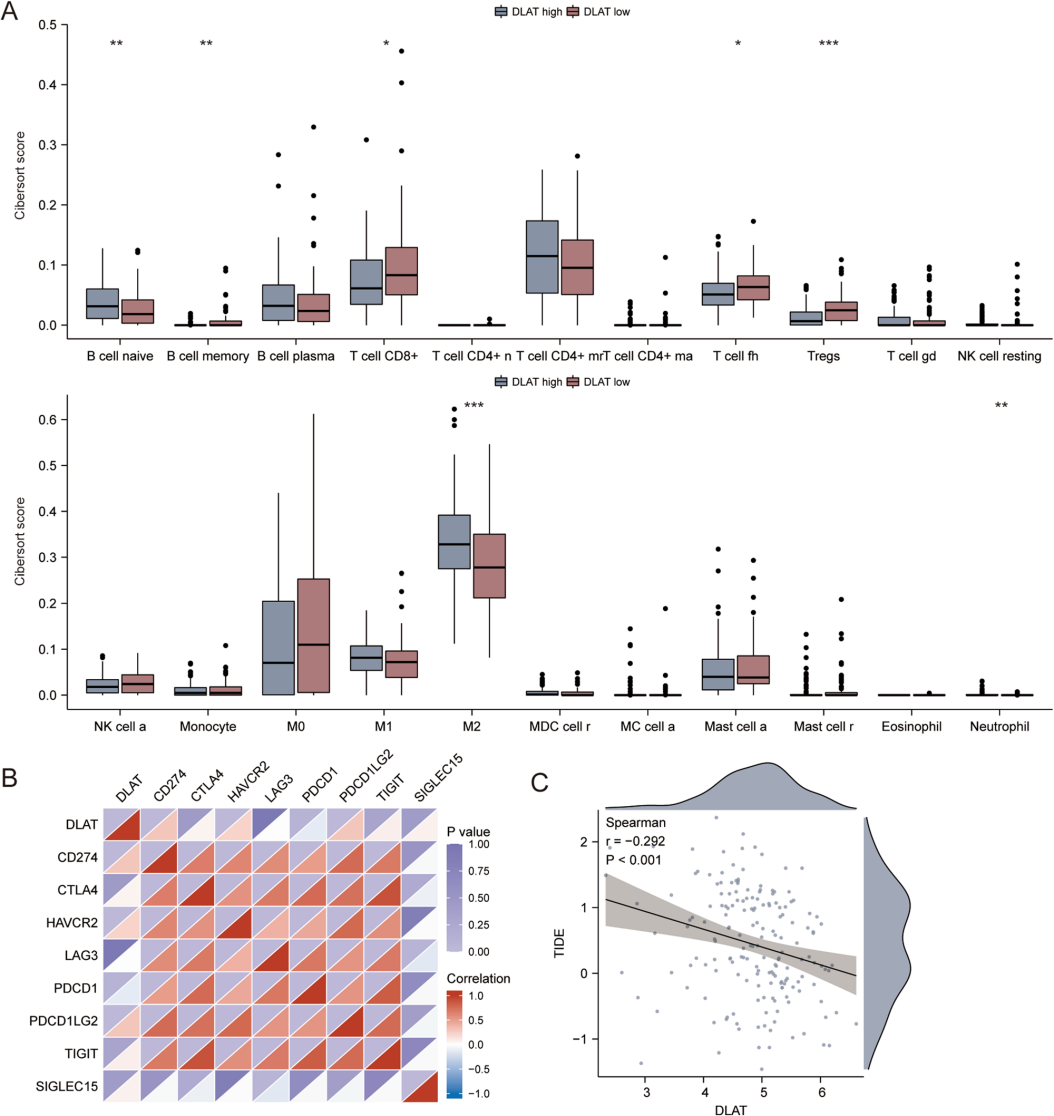
The immune landscape for *DLAT*. (**A**) The infiltrating levels of immune cells in high and low *DLAT* expression groups in HER2-positive breast cancer patients. *p < 0.05; **p < 0.01; ***p < 0.001. (**B**) The correlation between *DLAT* and immune checkpoint inhibitors related genes. (**C**) TIDE predicted response to immunotherapy.

We used the CIBERSORT algorithm to assess the difference of immune infiltration in HER2-positive BC patients with high or low *DLAT* expression. There is obvious difference in the distribution of 7 out of 22 immunocytes. As compared to the *DLAT* low group, the infiltration of B cell naïve (*p* < 0.01) and M2 macrophages (*p* < 0.001) subtypes was higher, while the infiltrating of B cell memory (*p* < 0.01), T cell CD8^+^ (*p* < 0.05), T cell fh (*p* < 0.05), Tregs (*p* < 0.001) and Neutrophil (*p* < 0.01) subtypes was lower in *DLAT* high group (Fig. 8A). We analyzed the correlation between *DLAT* and immune checkpoint inhibitors related genes (Fig. 8B). In addition, the difference in sensitivity to immunotherapy between patients in the *DLAT* high and low groups was further investigated using the TIDE algorithm. The TIDE algorithm is a recently developed tool for determining the efficacy of tumor immune checkpoint therapy^32^. In the present study, we found there was a negative correlation between TIDE and *DLAT* expression (r =  − 0.292, *p* < 0.001, Fig. 8C).

### The predicted value of *DLAT* for HER2-low BC patients

**Figure 9:**
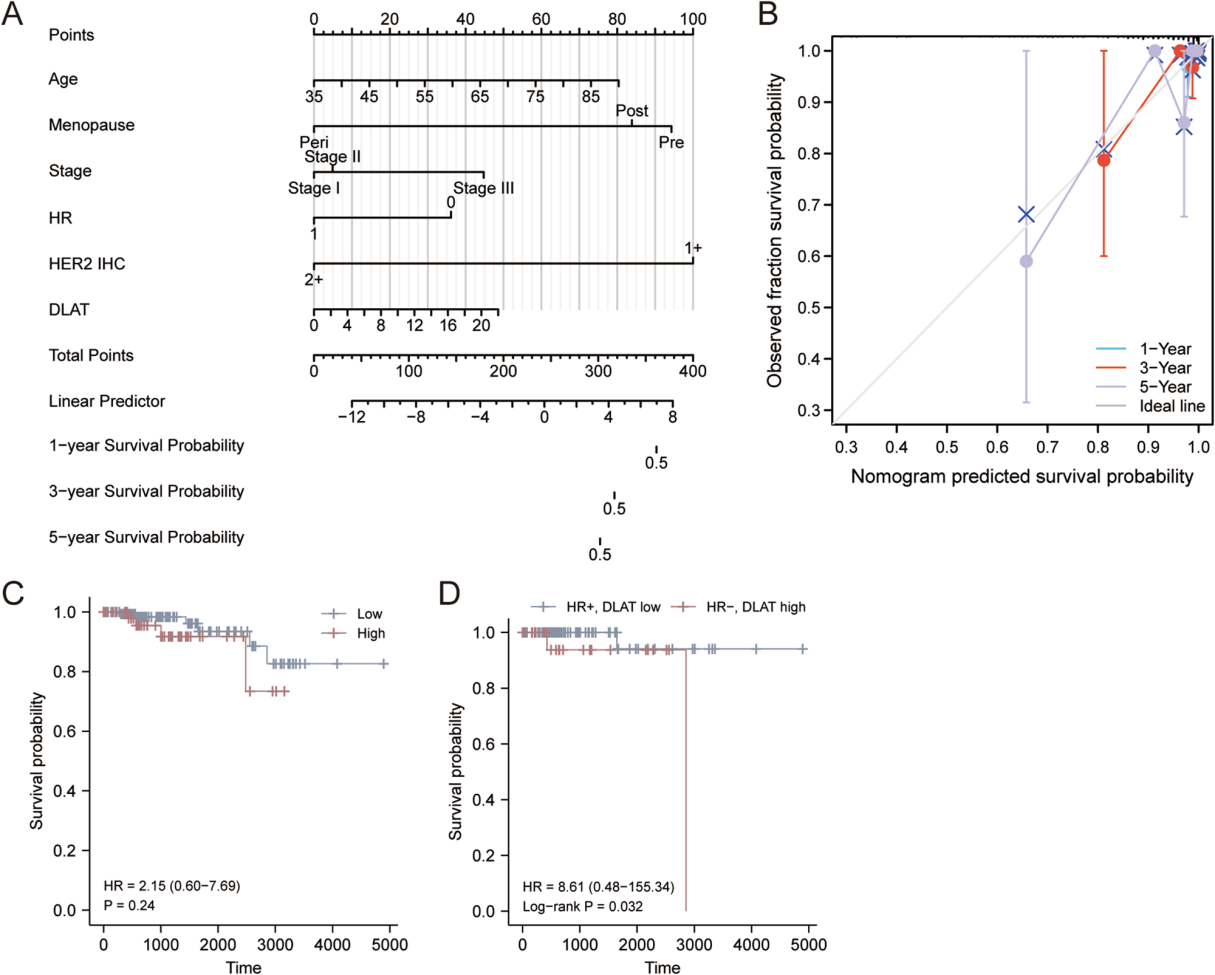
The predicted value of DLAT for HER2-low breast cancer patients. (**A**) The nomogram for predicting HER2-low breast cancer patients’ 1-, 2-, and 3-year OS probability. (**B**) The 1-, 2-, and 3-year calibration curves of the nomogram. (**C**) The OS curves of HER2-low breast cancer patients between DLAT low and high groups. (**D**) The OS curves of HER2-low breast cancer patients between HR+, DLAT low and HR−, DLAT high groups.

We further established the prognostic nomogram to quantitatively predict the 1-, 2- and 3-year survival probability of HER2-low BC patients (Fig. 9A). The calibration curve of the nomogram demonstrated that it could predict the survival probability relatively well (Fig. 9B). Although there had no difference of OS between DLAT high and low groups for HER2-low BC patients (HR = 2.15, *p* = 0.24, Fig. 9C). However, the HR+, DLAT low patients had longer OS than HR−, DLAT high patients (HR = 8.61, *p* = 0.032, Fig. 9D).

## Discussion

Cuproptosis is a newly defined RCD, which occurs via direct binding of copper to lipoylated components of the tricarboxylic acid (TCA) cycle and this results in lipoylated protein aggregation and subsequent iron-sulfur cluster protein loss leading to proteotoxic stress and ultimately cell death^19^. In this study, we identified a novel prognostic model consisting of four CRGs for HER2-positive BC patients. *DLAT* was downregulated and correlated with better survival of HER2-positive BC patients. High expressed *DLAT* was associated with resistant to HER2-targeted therapy. However, high expressed *DLAT* had lower TIDE score, which means it is sensitive to immunotherapy.

The prognostic model for HER2-positive BC was established according to the LASSO method. Four CRGs, named *ATOX1, DLAT, SLC31A2* and *SLC25A3*, were included in this prognostic model. *DLAT* gene was the E2 core component of the pyruvate dehydrogenase (PDH) mitochondrial enzyme complex, which encoding dihydrolipoamide acetyltransferase. Dihydrolipoamide acetyltransferase accepts acetyl groups and transfers them to coenzyme A^33,34^. *DLAT* plays an important role in glucose metabolism and TCA cycle^19,35^. Goh et al. found that *DLAT* had higher expression in eleven gastric cancer cells lines compared with non-cancer gastric epithelial cell line HFE145, and pyruvate level was increased when *DLAT* knockdown with siRNA^36^. Another research found that *DLAT* expression have positive relationship with PD-L1 in a clinical hepatocellular carcinoma (HCC) cohort, and *DLAT* was upregulated and correlated with poor prognosis^37^. Zhou et al. found that in the HCC cells, the proliferation and invasion ability was decreased once *DLAT* was knockdown, and the PI3K/Akt or Wnt/β-catenin signaling pathways were also significantly inhibited^38^. In our study, however, *DLAT* was downregulated in HER2-positive BC patients compared with normal tissues. Patients in the low *DLAT* expression group had significantly longer OS than patients in the high expression group. The negative correlation between *DLAT* expression and infiltrating of B cell memory, CD8^+^ T cell, fh T cell, Tregs and neutrophil was the unique immune landscape for HER2-positive BC. But a positive correlation between *DLAT* expression and B cells, CD4^+^ T cells, CD8^+^ T cells, neutrophils, macrophages and dendritic cells was found in the HCC cells^38^. The GSEA verified the potential roles of *DLAT* associated molecules in the regulation of cell cycle checkpoints, class I MHC mediated antigen processing presentation, DNA repair, interferon signaling and organelle biogenesis and maintenance. *DLAT* may play distinct roles between HCC and HER2-positive BC.

Several potential mechanisms, such as reduced drug binding to HER2, constitutive activation of HER2 parallel or downstream signaling pathways, metabolic reprogramming or reduced immune system activation, have been identified as primary/secondary resistance to anti-HER2 agents^14^. Trastuzumab can bind with HER2 extracellular domain IV, which can further prevent the formation of HER2-HER2 homodimers and HER2-HER3 heterodimers^39^, this also leads to the inhibition of HER2 ectodomain shedding and prevent the expression of the p95-HER2 isoform^40^. The additional mechanisms including antibody-dependent cellular cytotoxicity (ADCC) and complement-dependent cytotoxicity (CDC). Trastuzumab was recognized by Fc receptors of the innate immune system cells, including natural killer cells, antigen presenting cells and effector immune cells, once it bound to HER2 on cancer cell membranes, and this reduced the bounding of trastuzumab with cancer cell^41,42^. In our study, we found that *DLAT* high expressed HER2-positive BC patients have shorter OS and associated with resistance to trastuzumab and trastuzumab + pertuzumab. We identified *DLAT* had positive correlation with DNA replication and tumor proliferation and *DLAT* also had higher expression in T4 stage than T1&T2&T3 stage. All the evidence suggests that HER2-positive BC patients with high *DLAT* expression have a higher degree of malignancy and get limited benefits from HER2-targeted therapy. In our vitro experiment, knocking down of DLAT with siRNA significantly improved the treatment effect of trastuzumab. In addition, we found that high expressed *DLAT* had lower TIDE score, which means it is sensitive to immunotherapy. Immunotherapy has changed the treatment of several cancer types, and it has already been approved as a standard of care for patients with TNBC^43,44^. Immunotherapy for HER2-positive BC have been an interesting field and it is currently under clinical investigation. Based on the high TMB and high levels of tumor-infiltrating lymphocytes (TILs) of HER2-positive BC, investigating of immunotherapy for HER2-positive BC is feasible^45,46^. In this study, we found that *DLAT* also had significant positive correlation with TMB. *DLAT* high expressed HER2-positive BC patients may benefit from HER2-targeted therapy combined with immunotherapy.

Disruption of mitochondrial activity leading to physiological imbalances and metabolic shifts in the cell, which contribute to cancer progression. One of the most important reasons is mutations in TCA cycle enzymes^47,48^. However, as part of the TCA cycle enzymes, the somatic mutation rate of *DLAT* was 0.2% according to this study. The typical PDH deficiency patients were caused by E1α mutations, however, two PDH deficiency patients with *DLAT* mutation reported by Head et al. were less severely and both have survived well into childhood^49^. Shatokhina et al. demonstrated that *DLAT* was down regulated after the control U87 glioma cells were exposed under glutamine deprivation^50^. In our study, the expression level of *DLAT* had no difference between HER2-negative and HER2-positive patients. Nevertheless, it was still ambiguous whether *DLAT* was aberrant regulated in HER2-positive BC patients under some specific conditions. TCA cycle genes including *IDH2, SUCLA2, SDHB, SDHD* were downregulated after co-expression of HER2 and mucin 1 cytoplasmic domain (*MUC1-CD*). Interestingly, *MUC1-CD* induced reduction in TCA cycle genes was found to be significantly associated with poor survivals in patients with HER2-positive BC^51^. Chang et al. demonstrated that in trastuzumab resistant gastric cancer cell lines (NCI N87R and MKN45R), the activity of GATA binding protein 6 (*GATA6*) was enhanced prominently. In *GATA6* knockout cell lines, the sensitivity to trastuzumab was improved and the mitochondrial function was inhibited^52^. As *DLAT* plays an important role in glucose metabolism and TCA cycle, it also acted as a CRGs. In our study, KEGG pathway and GO enrichment of DEGs indicated that *DLAT* participates in various pathways correlate with organelle fission, chromosome segregation, nuclear division, hormone-mediated signaling pathway, regulation of intracellular estrogen receptor signaling pathway, condensed chromosome and PPAR signaling pathway. Despite the fact that the expression of *DLAT* had no difference between HER2-negative and HER2-positive BC patients, the exact function of *DLAT* in HER2-positive BC need further investigation.

In summary, we identified a novel prognostic model consisting of four CRGs for HER2-positive BC patients. In addition, *DLAT* was further confirmed to be downregulated and correlated with better survival of HER2-positive BC patients. High expressed *DLAT* related to resistance to HER2-targeted therapy and sensitivity to immunotherapy. However, this study also had some limitations. Firstly, the 14 CRGs were selected from the literature and there may exist some other genes related with cuproptosis, those results we got just applicable at the present stage. Secondly, we did not explore the detailed function of the four CRGs, especially *DLAT*, in HER2-positive BC. Furthermore, it is unclear how *DLAT* affects anti-tumor immunity in HER2-positive BC patients.

## Methods

### Data resource and procession

RNA sequencing data of BC and normal tissues and their corresponding clinical characteristics were obtained from The Cancer Genome Altas (TCGA, https://portal.gdc.cancer.gov/) and the Gene Expression Omnibus (GEO, https://www.ncbi.nlm.nih.gov/geo/). All patients included in this study were female. Subsequent analyses were performed with R software (v4.0.3). If not mentioned, *p* value < 0.05 were considered statistically significant.

### Expression analysis and survival analysis of prognostic CRGs in breast cancer

Fourteen genes related to cuproptosis were retrieved from the latest literature^30^. The differential expression of 14 CRGs between BC and normal tissues was compared with student t-test, including *ATOX1, CDKN2A, DLAT, DLD, FDX1, GLS, LIAS, LIPT1, MFT1, PDHA1, PDHB, SLC25A3, SLC31A1*, and *SLC31A2*. Univariate Cox regression analysis was performed to find genes with prognostic value in HER2-positive BC. Survival analyses were performed and Kaplan–Meier survival curves were constructed. The boxplot, forest plot and survival plot were carried out with R packages ‘ggplot2’, ‘forestploter’, and ‘suviminer’.

### The establishment of a CRG-based model and prognostic analysis

We established an efficient prediction model using LASSO-Cox analysis according to the methods mentioned in this article^53^. The risk score was calculated as follows: Riskscore = (0.1843) * *DLAT* + (− 0.4056) * *SLC31A2* + (0.7899) * *SLC25A3* + (0.4987) * *ATOX1*. The coefficient index represents the risk factor, and the gene name represents the expression level of each gene. According to the median risk score, the patients were divided into high risk (risk score > median) and low risk (risk score < median) groups. The LASSO regression, rank dot plot, scatter plots, heat map and survival plot were carried out with R packages ‘ggplot2’, ‘glmnet’, and ‘suviminer’.

### Differential expression genes (DEGs) in trastuzumab-resistant datasets

The anti-HER2 therapy datasets were derived from GSE121105 from GEO. We used three groups in this dataset: normal HER2-positive BC cell line (BT474), Trastuzumab resistant cell line (BT474-TR1, BT474-TR2), and both Trastuzumab and Pertuzumab resistant cell line (BT-TPR1, BT-TPR2). Each group contains 3 replicates of sequencing data. The DEGs were selected using the empirical Bayesian approach^54^. The significance criteria were set as an adjusted *p* value < 0.001 and an absolute value of Log_2_ Fold change > 1.5. The volcano plot and Venn diagram were carried out with R packages ‘GEOquery’, ‘limma’, ‘ggplot2’, and ‘VennDiagram’.

### The analyses of *DLAT*’s correlation with genetic characteristics

The genetic characteristics information was obtained from TCGA. The somatic DNA mutations of *DLAT* were downloaded and visualized using the R packages ‘maftools’. The DNA mismatch repair (MMR) was evaluated with the expression of 5 mismatching repair genes^55^. The Tumor mutation burden (TMB)^56^, Microsatellite instability (TSI)^57^, and cancer stemness score^58^ were calculated with Spearman’s correlation analysis. The genes related to the DNA replication, tumor proliferation and angiogenesis were picked up from the pathways mentioned in this article^59^ with R package ‘GSVA’ chosing parameter as method = ‘ssgsea’ and also analyzed by Spearman’s correlation. The scatter plot was carried out with R packages ‘ggstatsplot’.

### The analyses of *DLAT*’s correlation with clinical features

Based on TCGA data, the Kruskal–Wallis test was used to explore the relationships between *DLAT* and clinical features (ages, pTNM stages, PAM50 subtypes, menopause status, and biomarkers). The boxplot, Sankey diagram, and survival plot were carried out with R packages ‘ggplot2’, ‘ggalluvial’, and ‘suviminer’. The KM-plotter database^60^ was used to explore the replase free survival (RFS) of HER2-positive BC with different *DLAT* expression. TNM plotter database^61^ were used to explore the expression of DLAT in normal breast tissues, HER2-positive BC and metastatic tissues.

### The functional enrichment analyses of *DLAT*

HER2-positive BC samples in the TCGA dataset were divided by *DLAT* median expression to obtain DEGs. The significance criteria were set as an adjusted *p* value < 0.001 and an absolute value of Log_2_ Fold change > 1. The Gene Ontology (GO) and Kyoto Encyclopedia of Genes Genomes (KEGG)^62,63^ analyses were based on the DEGs. The Gene Set Enrichment Analysis (GSEA) of *DLAT* was further investigated. All the functional enrichment analyses and visualization were carried out with R packages ‘clusterProfiler’, ‘org.Hs.eg.db’, ‘msigdbr’, ‘GOplot’, and ‘ggplot2’.

### The immunological analyses of *DLAT* in the HER2-positive breast cancer microenvironment

The immune landscape of *DLAT* was first investigated by calculating by CIBERSORT algorithm^64^ by using R package ‘immunedeconv’. The immune checkpoint markers were extracted from a previous article^65^ and analyzed by Spearman’s correlation. The boxplot and heatmap were carried out with R packages ‘ggplot2’and ‘corrplot’. The potential response to immunotherapy was predicted by the Tumor Immune Dysfunction and Exclusion (TIDE) algorithm (http://tide.dfci.harvard.edu/)^32^. The scatter plot was carried out with R packages ‘ggstatsplot’.

### Confirming the prognostic value of *DLAT* in HER2-low patients

The HER2-low patients were defined as HER2 immunohistochemistry (IHC) results as “1+” in the TCGA dataset. The multivariate Cox regression and corresponding nomogram scoring system were used to develop predictive models of 1-, 3-, and 5-year survival rates. The calibration curve was used to compare the predicted survival rate with the actual one of 1-, 3-, and 5-year survival rates. The nomogram, calibration curve and survival curve were drawn by R package ‘survival’ and ‘rms’.

### Cell culture

The human breast cancer cell lines SKBR-3 was obtained from the American Type Culture Collection (ATCC), and cultured in RPMI-1640 medium (Gibco, USA) supplemented with 10% fetal bovine serum (Gibco, USA) and 1% penicillin/streptomycin (Sigma-Aldrich, USA) at 37°C in a humidified incubator with 5% CO_2_.

### Cell transfection

To knock down DLAT gene expression, small interfering RNA (siRNAs) were transfected into cell with jetPRIME (Polyplus-transfection Inc, France) according to the Manufacturer’s instructions. Prior to functional studies, we characterized and optimized the knock down of DLAT using 3 different siRNA sequences (si-DLAT-1, si-DLAT-2 and si-DLAT-3) over a concentration of 10, 25, 50 and 100 nM. 50 nM of siRNA was deemed to be optimal for knock down of DLAT. In addition, si-DLAT-1 and si-DLAT-2 were more effective in silencing DLAT expression than si-DLAT-3 (data not shown). The siRNA sequences were purchased from Life Technologies (Thermo Fisher Scientific, Rockford, IL) under the following product codes targeting the respective exons: si-DLAT-1 sequence (HSS102785, targeting exon 1): 5′-UCGCAACAGCGUGACUACAGGGUA-3′; si-DLAT-2 sequence (HSS102786, targeting exon 13): 5′-GGAUAAACUGGUCCCUGCAGAUAAU-3′. Luc control siRNA #5270471 was purchased from Life Technologies, USA.

### RNA extraction and RT-qPCR

Total RNA of cells was extracted using Simply P Total RNA Extraction kit (Bioer Technology, China) and subsequently reverse transcribed to complementary deoxyribonucleic acid (cDNA) using PrimerScript™ RT Master Mix (Takara, Shiga, Japan) on a SimpliAmp™ Thermal Cycler (Applied Biosystems, Massachusetts, USA) according to the manufacturer’s instructions. Obtained cDNAs were quantified with a reverse transcription quantitative polymerase chain reaction (RT-qPCR) test labeled with SYBR® green (Invitrogen, California, USA) on LightCycler® 96 (Roche, Mannheim, Germany). The gene-specific primers used were as follows: DLAT forward primer, 5′-CCGCCGCTATTACAGTCTTCC-3′, DLAT reverse primer: 5′-CTCTGCAATTAGGTCACCTTCAT-3′, β-actin forward primer, 5′-CATGTACGTTGCTATCCAGGC-3′; β-actin reverse primer, 5′-CTCCTTAATGTCACGCACGAT-3′. Gene expression levels were normalized to β-actin expression using the 2^−ΔCT^ method. Each cDNA sample was triplicated in 96-microwell plates.

### Cell growth assay

Growth inhibition was assessed using the 3-(4,5-Dimethylthiazol-2-yl)-2,5-diphenyltetrazolium bromide (MTT) Cell Proliferation and Cytotoxicity Assay Kit (Beyotime Institute of Biotechnology, Shanghai, China). Cells were diluted in 100 μl/well of maintenance cell culture media and plated in 96-well flat-bottom plates. The number of SKBR-3 cells per well used in the subsequent experiments were 5000. At 24 h after plating, cell culture media were replaced with 10% FBS-containing media with and without trastuzumab, followed by incubation for an additional 120 h. Trastuzumab concentrations ranged from 0.001 to 100 μg/ml. A total of 3 plate wells were set for each experimental point, and all experiments were carried out at least in triplicate. Data are expressed as percentage of growth relative to that of untreated control cells.

### Supplementary Information

Supplementary Information 1.

Supplementary Information 2.

Supplementary Information 3.

Supplementary Information 4.

Supplementary Information 5.

## Data Availability

Any data and R script in this study can be obtained from the corresponding author upon reasonable request. The final manuscript was read and approved by all authors. In this study, publicly available datasets were analyzed. These are available on The Cancer Genome Atlas (https://portal.gdc.cancer.gov/) and GEO (https://www.ncbi.nlm.nih.gov/).
