# Author Correction: Cuproptosis-related genes predict prognosis and trastuzumab therapeutic response in HER2-positive breast cancer

**DOI:** 10.1038/s41598-024-58710-7

**Published:** 2024-04-04

**Authors:** Rui Sha, Xinrui Dong, Shanshan Yan, Huijuan Dai, Aijun Sun, Liuxia You, Zongjin Guo

**Affiliations:** 1 Department of Thyroid and Breast Surgery, The First Affiliated Hospital of Wannan Medical College (Yijishan Hospital of Wannan Medical College), 2 Zheshan West Road, Wuhu, 241001 Anhui China; 2 Department of Breast Surgery, The First Affiliated Hospital With Nanjing Medical University, 300 Guangzhou Road, Nanjing, 210029 Jiangsu China; 3 Center for Medical Ultrasound, Suzhou Municipal Hospital, Nanjing Medical University Affiliated Suzhou Hospital, Suzhou, 215000 Jiangsu China; 4 Renji Hospital, School of Medicine, Shanghai Jiaotong University, 1630 Dongfang Road, Shanghai, 200127 China; 5 Department of Thyroid and Breast Oncological Surgery, Xuzhou Medical College Affiliated Huaian Hospital, 62 Huaihai South Road, Huaian, 223001 Jiangsu China; 6 Department of Clinical Laboratory, The Second Affiliated Hospital of Fujian Medical University, Quanzhou, 362000 Fujian China; 7 Department of Interventional Radiology, The University of HongKong-Shenzhen Hospital, Shenzhen, 518053 China

Correction to: *Scientific Reports* 10.1038/s41598-024-52638-8, published online 05 February 2024

The original version of this Article contained an error in the order of the author names, which was incorrectly given as Rui Sha, Xinrui Dong, Shanshan Yan, Huijuan Dai, Liuxia You, Zongjin Guo and Aijun Sun.

The original Article has been corrected.

