# Supplementary material for: Cuproptosis-related genes predict prognosis and trastuzumab therapeutic response in HER2-positive breast cancer
Source: Sci Rep. 2024 Feb 5;14:2908. doi: 10.1038/s41598-024-52638-8 (PMC10844230; doi:10.1038/s41598-024-52638-8)
Supplement: Supplementary file 1 — Supplementary Information 1. [file 41598_2024_52638_MOESM1_ESM.rtf]

Title£ºglmnt R
Legend: R packages script of 'glmnt',  and the supplementary material for Figure 3

library(glmnet)
fit <- glmnet(x,y,alpha=1)
coef(fit)
coefplot(fit)
